# The characteristics and countermeasures of coupled and coordinated development between technological innovation and ecological environment in China’s Gansu province

**DOI:** 10.1371/journal.pone.0290704

**Published:** 2023-10-03

**Authors:** Yongzhen Wang, Xianzhong Cao

**Affiliations:** 1 Urban Development Research Institute of Gansu, Lanzhou City University, Lanzhou, 730070, China; 2 Urban Development Research Institute of East China Normal University, Shanghai, 200062, China; Qufu Normal University, CHINA

## Abstract

The investigation of the coupling and coordination association between scientific and technological innovation and the eco-environmental system is of vital practical significance for promoting the high-quality economic development of China’s Gansu province. The evaluation index system is constructed based on the explanation of the coupling and coordination mechanism of a “Binary system”. Subsequently, a comprehensive evaluation model is developed using the entropy method, whereas the coupling coordination relationship between “Binary systems” is analyzed in the context of the coupling coordination degree model. The study findings indicate that the innovation drive has become the primary driving force that leads the high-quality economic development in Gansu province. Furthermore, the comprehensive development level and coupling and coordination degree of all systems in Gansu province demonstrate a sound trend of steadily rising. Additionally, there is an issue of uncoordinated development between scientific and technological innovation and eco-environment systems in Gansu province. Thus, this research study proposes certain policy suggestions, such as optimizing the environment of Sci-tech innovation, insisting on the priority of ecology, increasing the input of Sci-tech innovation, and building up a contingent of talents.

## 1. Introduction

Since the beginning of the 21st century, China’s social and economic development has gradually changed from high-speed growth to a high-quality development stage. Although, scientific and technological innovation exerts different driving effects on the ecological environment, under the conditions of different development stages and levels, and seriously influences the benign interaction between scientific and technological innovation and the ecological environment [1–4]. For instance, the report of the 19th National Congress of the CPC explicitly points out that “Innovation” represents the core status of high-quality development whereas“Green” serves as a crucial guarantee of high-quality development. Furthermore, scientific and technological innovation is helping to improve energy use efficiency, reduce ecological pollution, and develop a green market; thereby, playing a significant role in promoting the realization of a harmonious and green ecological environment. Parallel to this, the eco-environment offers substantial support for scientific and technological innovation by extending essential resources, talents, and the environment. Meanwhile, there exist complex relationships between these variables. Therefore, the current research hotspot of ecology, geography, and environment aims to realize the coupling and coordinated development of science and technology innovation and ecological environment, since it is of vital significance to the high-quality development and modernization drive of China.

Since the theory of innovation development was proposed in 1912 [5], therefore extensive research studies have been performed on regional innovation, with foreign studies mostly focusing on the influence of regional technological innovation on ecological environment optimization. Reportedly, Fussler and James put forward the notion of “ecological innovation,” that is, technological innovation is able to not only enhance production efficiency but also reduce the adverse impact of economic production activities on the ecological environment [6]. In the same vein, Barker et al. studied the classification and various impacts of technological innovation in different directions on ecological environment construction [7]. Moreover, Chen et al. adopted the input−output dimension to develop a Sci-tech innovation system and employed the DEA method to anticipate the effect and contribution of Sci-tech innovation efficiency on the regional eco-environment construction [8]. Besides this, Anadon et al. held that the backwardness of technological innovation is one of the major reasons behind environmental pollution, and the key to resolving environmental concerns is to enhance the level of technological innovation [9]. Owing to the growth of industrialization and urbanization, environmental pollution has become increasingly serious, therefore the government authorities need to incorporate relevant environmental regulations, which would certainly impact regional technological innovation and production efficiency [10]. In addition, the “Baud hypothesis” illuminates that strict environmental regulation modifies the production model of enterprises in the long run, stimulates the vitality of firms’ technological innovation, and eventually attains the benefits of innovation and first-mover advantage [11]. Furthermore, Guo et al. stressed that the ecological environment extends the sustainable basis of resources, materials, and space for technological innovation, while the key is to coordinate the connection between them, in order to effectively realize regional green development [12]. At the same time, Zhang et al. emphasized that environmental regulation not only increases the cost of corporate governance but also makes the enterprises’ capital shift the investment of innovation projects to pollution control measures; thus, reducing the enthusiasm of enterprises for scientific and technological innovation [13].

Certain domestic studies were performed, and some accomplishments were made at the beginning of the new century. Based on the dimension of coercion and restriction, various studies asserted that technology innovation heavily relies on the resources and environment in the extensive stage, which would undeniably uplift the burden of the regional ecological environment [14]. In particular, the prompt adoption of environmental regulation stimulates innovation practices and lowers the cost and resource dependence of innovation in the extensive stage [15]. Additionally, there exists a certain synergy relationship as a significant positive influential mechanism has been established between technological innovation and environmental pollution control [16]. From the standpoint of preference and technological innovation, several studies deduced that technological innovation is able to restrain the emission of industrial “three wastes” while effectively promoting the regional environmental governance level [17]. Another study examines the effect of technological innovation on regional energy use’s intensity through the space Dubin model; hence, emphasizing the influence of technological innovation on the intensity of regional energy use and spatial spillover effect [18]. Besides, various research studies ascertained the evolution law of the coupling and harmonious development of technological innovation and ecological environment optimization. From the perspective of time-series change, different studies adopted the entropy weight method and the coupling coordinated development degree model to highlight the time-series attributes of the coupling coordinated development of technological innovation and ecological environment in China; consequently, emphasizing the continuous improvement in the development level of these two variables and rendering prominent the regional differences [19]. Supported by the coefficient of variation and the coordination degree model, a large number of studies assumed that the spatial distribution pattern of the coordination degree of technological innovation and ecological environment in China presents the narrative of “east high, east low, west low” [20].

In short, although there is abundant research on the association between scientific and technological innovation and eco-environmental optimization, most of these studies are confined to the one-way effects between these two variables. In terms of the coupling between scientific and technological innovation and eco-environmental optimization, the coordinated development of research is still not only relatively less plaque but also lacks the coupling of the coordinated development of the space-time characteristics of the analysis. In addition to this, the spatial spillover effect between regions is seldom taken into account in the analysis of driving factors in the extant literature; resultantly, ignoring the interactions between different regions may lead to a quantitative analysis bias in the drivers. Based on the lack of research in the existing literature, this research paper takes Gansu province as the research object, attempts to disclose the coupling and coordination mechanism between scientific and technological innovation and the ecological environment, and builds the Comprehensive Evaluation Index system of both. Moreover, the panel data of Regional Science and technology innovation and ecological environment in Gansu province are analyzed for the time period from 2015 to 2019 by using the entropy method and coupling coordination degree model, since it can not only improve the quality of economic development in the less-developed regions of western China but also support the successful transformation of the area into a domestic second-class urban agglomeration. At the same time, it also constitutes the basis and reference for devising the strategy of ecological civilization and internal coordinated development in western China; thus, offering valuable reference for other less-developed regions.

## 2. Theoretical analysis and research design

### 2.1 The connotation of ecological innovation theory

“Ecological innovation” is also often referred to as“Green innovation, “Sustainable Innovation”, and “Environmental innovation”. The concept of sustainable development can be traced back to the United Nations in 1972. Notably, Fussler and James first proposed the notion of“Ecological innovation”[21], which was refined and enriched by various organizations and research scholars such as the European Union’s measuring eco-innovation project (Mei), the OECD’s DECD and Dong Ying [22–24]. This implies that ecological innovation should not only include the innovation of front-end technology and methods, the sale of novel products and processes in the middle-end but also environmental governance in the end. Alternatively, the environmental and ecological benefits should not be neglected while economic benefits must be emphasized, therefore, ecological innovation comprises the main engine to achieve high-quality regional development. This research article constructs a comprehensive evaluation index system of regional scientific and technological innovation and ecological environment in Gansu Province, in order to analyze the coupling degree, the coordinated development level, and the spatial-temporal pattern difference between the two. Afterward, a comprehensive evaluation index system of regional scientific and technological innovation and ecological environment is put forward, in order to extend rational suggestions for high-quality regional development of Gansu province.

### 2.2 The coupling and coordination mechanism between science and technology innovation and ecological environment in the context of regional high-quality development

#### 2.2.1 Analysis of the coupling and interaction mechanism between technological innovation and ecological environment from the perspective of high-quality regional development

The enterprises, educational institutes, and regional governments promote the rapid flow and circulation of innovative elements in the region by investing in innovative elements such as talents, funds and resources, information networking, and convenient transportation, in order to accelerate the pace of scientific and technological innovation, production mode and innovation of original technology; thus, facilitating the improvement in production efficiency [4,25]. Meanwhile, the green demand of the customers, the competitive pressure of the market, and the cost pressure of the enterprise promote the ecological innovation of the technology and support the production of new technology, products, and management methods. Consequently, scientific and technological innovation guarantees the application and spread of new and clean energy, reduces energy consumption, improves energy efficiency, and controls waste and pollutant emissions. Consistent with this technological innovation also promotes the use of eco-innovation products and the development of green markets; thereby, realizing the low-carbon environmental transformation of markets and enterprises, lowering the disorderly competition and waste of resources, and raising the threshold of market access. Similarly, this innovation also promotes the effectiveness and long-term end-of-environment governance while effectively preventing environmental degradation and pollution, in order to achieve environmental optimization and protection [26].

Owing to the long-term orientation, complexity, and diversity of ecological and environmental concerns, the government is expected to implement environmental regulations, supported by a series of policy incentives and various policies, including environmental protection laws and regulations, energy conservation and emission reduction, pollution charges, and environmental protection initiatives, in order to compel corporations to change their traditional mode of production, and maintain a sound balance of regional man-land relations, and upgrade their existing production technologies. Simultaneously, a sound ecological environment extends sustainable resources and industrial benefits for scientific and technological innovation, which is conducive to stimulating the ecological transformation of industrial structure and the concentration of innovative factors and improving the efficiency and productivity of scientific and technological innovation. Additionally, the process of continuous optimization of the ecological environment, exerts an influence on the regional factor endowment and industrial structure, while increasing the competitive pressure and external costs of enterprises. As a result, different regions promote S&T Innovation through optimal resource allocation and policy incentives [27,28].

**Fig 1:**
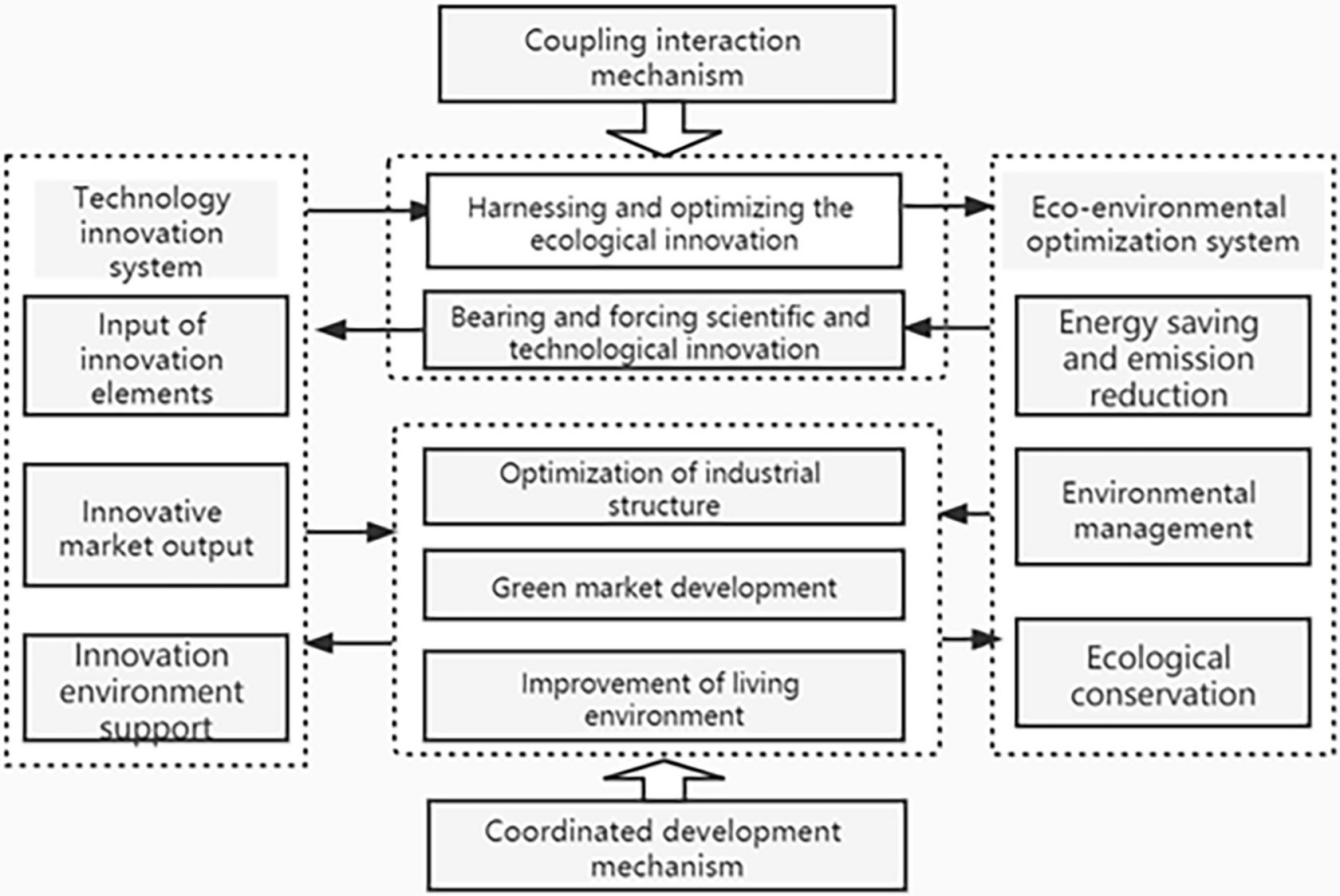
The coupling and coordination mechanism between regional technology innovation and regional ecological environment optimization.

Based on this, sound regional science and technological innovation is conducive to encouraging the governance and optimization of the ecological environment. Further, a sound regional ecological environment shall strengthen the ecological transformation of scientific and technological innovation (Fig 1), since these variables are associated with each other, represent influential mechanisms of coupling and interaction, and jointly push the region towards high-quality production and ecological transformation.

#### 2.2.2 Analysis of mechanism for coordinated development of scientific and technological innovation and eco-environmental optimization in the context of high-quality regional development

It is evident that there exists a strong dynamic correlation between regional scientific and technological innovation and the ecological environment, through the analysis of the interaction mechanism between the two systems. Moreover, it is highly significant to realize the coordinated development of the two systems from disorder to order. At the same time, the development of the two systems will restrict each other when there is a significant difference in the level of mutual development between the two systems; resultantly, hindering the optimization and upgrading of industrial structure, the improvement in living environment, and the development of the green market. Particularly, the coordinated development of two regional systems deviates from the ideal state [29]. Based on the aforementioned analysis, it is obvious that the coordinated development of the two systems not only involves the industry and market but also warrants the government’s effective regulation and suitable living environmental support.

### 2.3 Research methods

#### 2.3.1 Construction of the index system

A scientific and sound evaluation index system serves as the premise and basis for exploring both coupling of the regional technology innovation and ecological environment optimization. Based on the mechanism of the coupling- and harmonious development of the two systems, a comprehensive evaluation index system is constructed for both systems. In terms of the Comprehensive Evaluation Index System of regional technology innovation, this study follows Guo et al.’s construction of a comprehensive evaluation index system of regional S&T innovation from three levels namely: “innovation factor input−innovation market output−innovation environment support” [30]. Consistent with the index system of regional eco-environment optimization and Cheng et al. [31] and Wang et al. [32], the eco-environment optimization system is categorized into three different levels—energy-saving and emission reduction, ecological conservation, and environmental governance. Accordingly, Table 1 demonstrates the specific element indicators.

**Table 1:** Index system of technology innovation and ecological environment comprehensive evaluation.

Primary Index	Secondary Index	Tertiary Index	Unit	Weight	Attribute
A1: Technology Innovation	B1: Factor Input	C1:The NUMBER OF UNITS WITH R&D Activity	One	0.0977	+
		C2: The R&D Personnel are Equivalent to Full-Time Employees	People	0.1603	+
		C3: Internal Expenditure of R&D Funds	10,000 Dollars	0.1371	+
	B2: Market Output	C4: Technology Market Turnover	Billion	0.0944	+
		C5: Percentage of Patents Granted	%	0.0494	+
		C6: Innovation Expenses of Industrial Enterprises	Billion	0.0951	+
	B3: Environmental Support	C7: Percentage of Science and Technology Expenditure	%	0.1004	+
		C8: Average Number of Students per 100,000 Persons in Institutions of Higher Learning	One	0.1303	+
		C9: Number of R&D Institutions	One	0.1353	+
A2: Optimization of Ecological Environment	B4: Energy-Saving and Emission Reduction	C10: 10,000 Yuan GDP Industrial Waste Gas Emissions	10,000 Tons	0.0270	–
		C11: 10,000 Yuan GDP Industrial Waste Water Discharge	10,000 Tons	0.0180	–
		C12: Industrial Solid Waste Generation	10,000 Tons	0.0604	–
	B5: Ecological Conservation	C13: Per-capita Area of Parks and Green Spaces	Square Meter	0.1478	+
		C14: Green Coverage of Built-Up Area	%	0.0610	+
		C15: Per-capita Construction Land Area	Square Meter per Person	0.1993	+
	B6: Environmental Management	C16: Domestic Refuse Removal Capacity	10,000 Tons	0.2305	+
		C17: Number of Wastewater Treatment Facilities	Set	0.0853	+
		C18: Comprehensive Utilization of Industrial Solid Waste	10,000 Tons	0.1707	+

#### 2.3.2 Weighting method: The variation coefficient weighting method

The concrete measurements steps incorporated in this research study are as follows:

Firstly, the index is standardized to 0−1. The estimation expression is as follows:

$$x_{ij}^{'}=\{\begin{array}{l} \frac{x_{ij}-x_{j_{\min}}}{x_{j_{\max}}-x_{j_{\min}}}(1)\\ \frac{x_{j_{\max}}-x_{ij}}{x_{j_{\max}}-x_{j_{\min}}}(2) \end{array}$$

(1) *x*_*i*_ represents a positive indicator; (2) *x*_*i*_ stands for a negative indicator where $x_{ij}^{'}$ denotes the index value of item J after normalization; *x*_*ij*_ denotes the index value of item J; $x_{j_{\min}}$ connotes the minimum value of item J, whereas $x_{j_{\max}}$ shows the maximum value of item J. Afterward, the coefficient of variation *v*_*j*_ for each of the standardized indicators are measured as follows:

$$v_{j}=\frac{\partial_{i}}{{\bar{x}}_{j}},j=1,2,3\ldots m$$

where ${\bar{x}}_{j}$ indicates the average value of each standardized index, while ∂_*i*_ means the standard deviation. Finally, each index weight *w*_*j*_ is scaled as follows:

$$w_{j}=\frac{V_{j}}{\sum_{j=1}^{m}V_{j}},\;j=1,2,3\ldots m$$

#### 2.3.3 Coupled coordination degree model

The coupling formula stands as follows:

$$C_{i}=2\sqrt{\frac{A_{1i}\times A_{2i}}{\left(A_{1i}\times A_{2i})\times (A_{1i}\times A_{2i}\right)}}$$

where ***C***_***i***_ presents the coupling degree, with the value range as [0,1]; ***A***_***1i***_ stands for the science and technology innovation index of City I; and ***A***_***2i***_ serves as the ecological environment optimization index of City I.

The mathematical expression of the coupling coordination degree is below:

$$D_{i}=\sqrt{A_{1i}\times A_{2i}},\;T_{i}=\alpha A_{1i}+\beta A_{2i}$$

where ***D***_***i***_ signifies the degree of coupling coordination; ***α*, *β*** reflect the undetermined coefficient of scientific and technological innovation and ecological environment optimization, and *α +β =* 1. Besides this, *α* and *β* are assigned to 0.5 since scientific and technological innovation, and ecological environment optimization is at the same important level. Simultaneously, this paper classifies the proposed coupling coordination degree into 4 types (Table 2) based on the criterion of coupling degree of coordination proposed by Guo Aijun and other research scholars [30].

**Table 2:** Types of coupling coordination degree between scientific and technological innovation and ecological environment optimization.

*D*_*i*_ value	0.1–0.3	0.3–0.5	0.5–0.7	0.7–1.0
Type of Coupling Coordination	Weak Coupling Coordination	Moderate Coupling Coordination	Good Coupling and Coordination	High-Quality Coupling Coordination

### 2.4 Data sources

The original data used in this research is directly derived from the Gansu Development Almanac, China Energy Statistical Almanac in 2016−2020, and Gansu Science and Technology Statistical Almanac. Among them, the average interpolation method is employed to complete the data for the missing data of individual city years, in accordance with the values of adjacent years. Furthermore, the missing data of the C12 index is reported by three exponential smoothing algorithms using Excel software.

## 3. Empirical analysis

Using the entropy method and coupling coordination model, the researchers analyzed the situation of science and technology innovation, ecological environment optimization, and their coupling in 14 cities of China’s Gansu province. In addition to this, the coupling degree of scientific and technological innovation, ecological environment optimization, and the coupling degree of coordination is obtained by computing the coupling degree’s mathematical expression (Tables 3 and 4).

**Table 3:** Gansu province cities technology innovation and ecological environment optimization coupling degree estimation results during 2015−2019.

Cities	2015	2016	2017	2018	2019
Lanzhou City	0.9989	0.9936	0.9946	0.9955	0.9900
Jiayuguan City	0.8956	0.9329	0.8674	0.8083	0.8995
Jinchang City	0.9810	0.9946	0.9954	0.9486	0.9633
Baiyin City	0.8063	0.8451	0.8386	0.8361	0.8610
Tianshui City	0.8889	0.9340	0.9431	0.9651	0.9767
Wuwei City	0.9251	0.9569	0.9327	0.9552	0.9912
Zhangye City	0.8348	0.8398	0.8720	0.9727	0.9610
Pingliang City	0.6618	0.7006	0.6983	0.7281	0.7653
Jiuquan City	0.9008	0.9679	0.9342	0.9585	0.9398
Qingyang City	0.8374	0.8286	0.8454	0.7934	0.8569
Dingxi City	0.5850	0.6279	0.7314	0.7368	0.7437
Longnan City	0.7026	0.8436	0.8617	0.8796	0.8062
Linxia City	0.4102	0.2831	0.6423	0.7093	0.8288
Gannan Tibetan Autonomous Prefecture	0.8210	0.8323	0.7335	0.8179	0.7236

**Table 4:** Gansu province cities technology innovation and ecological environment optimization coupling coordination calculation results during 2015−2019.

Cities	2015	2016	2017	2018	2019
Lanzhou City	0.7740	0.7587	0.7581	0.7880	0.8421
Jiayuguan City	0.5095	0.5445	0.5532	0.5137	0.5718
Jinchang City	0.4886	0.4821	0.4899	0.4466	0.4591
Baiyin City	0.4009	0.4122	0.4125	0.4368	0.4680
Tianshui City	0.4012	0.4162	0.4179	0.4417	0.4557
Wuwei City	0.4001	0.4124	0.4298	0.4178	0.4542
Zhangye City	0.4969	0.5178	0.5427	0.4928	0.5005
Pingliang City	0.3294	0.3552	0.3424	0.3558	0.3770
Jiuquan City	0.4417	0.4454	0.4288	0.4408	0.4455
Qingyang City	0.3371	0.3195	0.3190	0.2974	0.3495
Dingxi City	0.2643	0.2843	0.3058	0.3266	0.3253
Longnan City	0.2547	0.2695	0.2913	0.2912	0.3168
Linxia City	0.1976	0.1615	0.2551	0.2749	0.3373
Gannan Tibetan Autonomous Prefecture	0.2618	0.2749	0.2522	0.2704	0.3037

A prominent coupling correlation exists between technological innovation and the ecological environment. As evident in Table 3, the coupling degree of technology innovation and ecological environment of 14 cities in Gansu province is at a high level, with the average value of coupling degree as 0.8791 in 2019, specifically the level of Lanzhou exceeded 0.9900. This implies that the Gansu province attaches great significance not only to economic growth driven by technological innovation but also to the protection of the ecological environment during development, which constitutes the core requirement and connotation of high-quality development. Nonetheless, the coupling degree of most cities exhibited a declining trend during 2015−2019. Since there are two major reasons behind this: (i) Firstly, Gansu province has excessively focused on the development of technology innovation in recent years. Besides, the investment in the ecological environment has been reinforced, but the advantage of environment protection has either lagged or shall be reflected on a medium and long-term basis; (ii) Secondly, the input of the ecological environment has been strengthened, but the benefits of eco-environmental protection have been lagged or shall demonstrate their effect in the long run.

Meanwhile, Tables 4 and 5 reflect the following characteristics. Primarily, the coupling and coordinated development level of technological innovation and eco-environmental optimization between the provincial capital and other cities in Gansu province project an overall upward trend from 2015 to 2019. Similarly, the mean value of the coupling coordination degree of the two systems in Gansu province is maintained between 0.2453 and 0.7842. Meanwhile, the value of the coupling coordination degree of the two systems in other states and cities records an increasing trend except for Jinchang, but the development pace is highly slow. Parallel to this, cities (states) in moderate coupling and coordination accounted for a comparatively large proportion of 57%. This reflects that the coupling and coordinated development level of the two systems as a whole in Gansu province presents a moderate coupling and coordinated development level. The proposed development situation is in line with the development situation in the eastern, central, and western regions of China.

**Table 5:** 2015–2019 coupling coordination degree and types of science and technology innovation and eco-environment optimization in Gansu province.

Cities	2015	Type	2019	Type	5-YEAR average	Type
Lanzhou City	0.7740	High-Quality Coupling Coordination	0.8421	High-Quality Coupling Coordination	0.7842	High-Quality Coupling Coordination
Jiayuguan City	0.5095	Good Coupling and Coordination	0.5718	Good Coupling and Coordination	0.5385	Good Coupling and Coordination
Jinchang City	0.4886	Moderate Coupling Coordination	0.4591	Moderate Coupling Coordination	0.4733	Moderate Coupling Coordination
Baiyin City	0.4009	Moderate Coupling Coordination	0.4680	Moderate Coupling Coordination	0.4261	Moderate Coupling Coordination
Tianshui City	0.4012	Moderate Coupling Coordination	0.4557	Moderate Coupling Coordination	0.4265	Moderate Coupling Coordination
Wuwei City	0.4001	Moderate Coupling Coordination	0.4542	Moderate Coupling Coordination	0.4229	Moderate Coupling Coordination
Zhangye City	0.4969	Moderate Coupling Coordination	0.5005	Good Coupling and Coordination	0.5101	Good Coupling and Coordination
Pingliang City	0.3294	Moderate Coupling Coordination	0.3770	Moderate Coupling Coordination	0.3520	Moderate Coupling Coordination
Jiuquan City	0.4417	Moderate Coupling Coordination	0.4455	Moderate Coupling Coordination	0.4404	Moderate Coupling Coordination
Qingyang City	0.3371	Moderate Coupling Coordination	0.3495	Moderate Coupling Coordination	0.3245	Moderate Coupling Coordination
Dingxi City	0.2643	Weak Coupling Coordination	0.3253	Moderate Coupling Coordination	0.3013	Moderate Coupling Coordination
Longnan City	0.2547	Weak Coupling Coordination	0.3168	Moderate Coupling Coordination	0.2847	Weak Coupling Coordination
Linxia city	0.1976	Weak Coupling Coordination	0.3373	Moderate Coupling Coordination	0.2453	Weak Coupling Coordination
Gannan Tibetan Autonomous Prefecture	0.2618	Weak Coupling Coordination	0.3037	Moderate Coupling Coordination	0.2726	Weak Coupling Coordination

Secondly, the coupling and coordinated development of technological innovation and eco-environmental optimization between the provincial capital city and other cities in China’s Gansu province is prominent in 2015−2019. Additionally, there exists a significant gap between the levels of eco-environmental optimization in Lanzhou Province. The level of coordinated development of scientific- and technological innovation and eco-environmental optimization in Gansu province is maintained at above 0.75, with the level of coordinated development of scientific and technological innovation and eco-environment optimization in other cities at less than 0.6, which demonstrates the substantial potential for the future. Moreover, there is a difference of 0.5764 between the highest and lowest level of urban eco-environmental optimization in 2015, whereas this difference reached 0.5384 in 2019. Noticeably, such development is consistent with the principle of coupling and coordinated development of scientific and technological innovation and ecological environment optimization in the underdeveloped regions of western China.

Third, the trend of coordinated development of technological innovation and ecological environment optimization demonstrates differentiation in 2015−2019 in the cities of Gansu province. In addition to this, the coordinated development level of technological innovation and ecological environment optimization in Linxia City has enhanced from 0.1976 to 0.3373 in 2015−2019, with an increment of 70.64%. Further, the coordinated development level of technological innovation and the ecological environment optimization in Jiuquan City has fluctuated up and down from 0.4371. Parallelly, the amplitude is not more than 0.015, which is relatively stable, while the coordinated development level of technological innovation and ecological environment optimization has exhibited a declining trend in Jinchang, from 0.4886 in 2015 to 0.4591 in 2019, with a decline of 6.03%.

Fourthly, the ranking of the coordinated development level of technological innovation and ecological environment optimization of cities in Gansu province has changed during 2015−2019. Specifically, Lanzhou, Jiayuguan City, Zhangye, Tianshui, and Longnan remained unchanged in terms of their rankings. However, Jinchang, Qingyang, and Dingxi moved one place up, with Gannan moving up two places. Lastly, Jiuquan moved up three places, with Baiyin, Gannan, Gannan, and Gannan being slipped in their positions.

## 4. Analysis of the subsystem coupling and coordination

In order to scrutinize the coupling and coordination association between technological innovation and the ecological environment, this paper has analyzed the coupling and coordination of subsystems (Tables 6 and 7).

**Table 6:** High degree of coupling coordination of the two-level indicator combination during 2015−2019.

Serial Number	Secondary Indicators of Technological Innovation	Secondary Indicator of Ecological Environment	Numerical Value
1	Factor Input	Environmental Management	0.6401
2	Factor Input	Energy-Saving and Emission Reduction	0.4280
3	Factor Input	Ecological Conservation	0.4516
4	Market Output	Energy-Saving and Emission Reduction	0.3353
5	Market Output	Environmental Management	0.4756
6	Market Output	Ecological Conservation	0.3538
7	Environmental Support	Energy-Saving and Emission Reduction	0.3904
8	Environmental Support	Environmental Management	0.5838
9	Environmental Support	Ecological Conservation	0.4118

**Table 7:** Three-level Index combination with higher coupling coordination degree during 2015−2019.

Serial Number	Three-Level Indicators of Technological Innovation	Three-Level Index of Ecological Environment	Numerical Value
1	R&D is a Full-Time Job	Domestic Refuse Removal Capacity	0.6929
2	Internal Expenditure of R&D Funds	Domestic Refuse Removal Capacity	0.6928
3	Technology Market Turnover	Domestic Refuse Removal Capacity	0.6674
4	Percentage of Patents Granted	Comprehensive Utilization of Industrial Solid Waste	0.3859
5	Percentage of Patents fed	Number of Wastewater Treatment Facilities	0.5052
6	Innovation Expenses of Industrial Enterprises	Per-Capita Area of Parks and Green Spaces	0.5016
7	Innovation Expenses of Industrial Enterprises	Per-Capita Construction Land Area	0.6230
8	Innovation Expenses of Industrial Enterprises	Number of Wastewater Treatment Facilities	0.4669
9	Innovation Expenses of Industrial Enterprises	Comprehensive Utilization of Industrial Solid Waste	0.4733
10	Percentage of Technology Expenditure	Per-Capita Construction Land Area	0.6137
11	Average Number of Students per 100,000 Persons in Institutions of Higher Learning	Domestic Refuse Removal Capacity	0.6929
12	Number of R&D Institutions	Domestic Refuse Removal Capacity	0.6660

Tables 6 and 7 illustrate that technological innovation extends different degrees of development support for environmental and ecological protection. In terms of the coupling and coordination degree between technological innovation and secondary indicators of the ecological environment, the secondary indexes with higher coupling coordination degree comprise factor input and energy-saving and emission reduction (0.4280), factor input and environmental management (0.6401), factor input and ecological maintenance (0.4516), market output and energy-saving and emission reduction (0.3353), market output and ecological maintenance (0.3538), market output and environmental management (0.4756), environmental support and energy-saving and emission reduction (0.3904), environmental support and ecological maintenance (0.4118), and environmental support and environmental management (0.5838). Among them, the coupling degree of factor input and environmental governance is the highest, followed by environmental support and environmental governance, market output, and energy-saving emission reduction, whereas ecological maintenance is the lowest. Besides, the protection and management of the ecological environment majorly benefitted from the support of an innovative environment and the input of technological innovation. Specifically, different cities in Gansu province have augmented their efforts in innovation environment and factor input, which is the foremost reason for surging the coupling degree of technological innovation and ecological environment.

Moreover, the coupling degree of internal expenditure of funds and three-level indexes, such as technology market turnover, domestic waste transportation volume, and domestic waste transportation volume, is relatively high, demonstrating that the improvement in eco-environmental protection and governance capacity in Gansu province highly associated with R&D personnel and investment. In addition, the transformation of technological achievements determines the extent of eco-environmental protection and governance to a certain extent; thus, providing strong support for Gansu province to materialize high-quality development programs in the future.

## 5. Conclusions and recommendations

### 5. 1 Research conclusion

From the perspective of system theory, this research article analyzes the coordinated development between science and technology innovation and the eco-environmental system in Gansu province by employing the ecological innovation theory and coupling coordination theory. Consequently, this research study draws the following conclusions:

Firstly, innovation-driven leads the high-quality economic development in China’s Gansu province as the first power. In terms of the growth rate of coupling coordination degree, Gansu province shall further enhance investment in science and Technology Innovation for a time period from 2015 to 2019. Besides this, both the growth trend of coupling coordination degrees, and the leading role of science and technology innovation are prominent.

Secondly, the comprehensive development level and coupling coordination degree of all systems in Gansu province exhibits a sound trend of steady uplift. The development level of both high-quality development binary integrated systems and each sub-system has been greatly improved over the period. Meanwhile, the degree of coupling coordination among all types of systems has developed from weak coupling coordination to moderate coupling coordination or sound coupling coordination.

Third, there exists an uncoordinated development between scientific and technological innovation and the eco-environmental system in Gansu province. Similarly, the development of scientific and technological innovation and the eco-environment system is out of step and harmony. The eco-environment system serves as the short board that restricts the high-quality development of the economy in the Gansu province of China.

### 5. 2 Policy suggestions

The Gansu province of China, as an imperative province in the Yellow River basin and western development, must realize the transformation and upgrading of“Ecology first, green development”. For this purpose, the coordinated and balanced development of each system is essential. Accordingly, the following research recommendations are proposed in this regard:

Firstly, there is a need to constantly optimize the environment for technological innovation. Therefore, Gansu province should create promising conditions in terms of not only policy and system but also from the viewpoint of finance and culture, in order to optimize the environment for technological innovation. Hence, China should not only rely on its strength to achieve innovation but also seize the potential opportunities to learn from advanced concepts and experiences at home and abroad, while reinforcing the collaborative innovation between technological enterprises, research institutions, local governments, and universities; thereby, giving full play to the leading innovation role of large-scale scientific and technological enterprises. In addition to this, small- and medium-scale enterprises should be supported to realize the sound development of industries, universities, and research institutions to make more effective use of talents and resources and form synergy to hasten the pace of innovation.

Secondly, relevant input should be continuously increased in technological innovation and the ecological environment. Together with the actual development of cities in Gansu province, there is a need to further increase the investment in both to avoid the synchronized development of the dual system led by technological innovation. In addition to this, there is a need to increase investment in R&D personnel and investment in R&D funds and devise medium- and long-term development plans to establish a robust and synchronized development between technological innovation and ecological and environmental protection. Meanwhile, policy support should be fortified to technology innovation and ecological environment protection in order to reduce the development gap between regions and attain the goal of balanced regional development.

Thirdly, ecological priority should be given optimum consideration to regional carrying capacity while adhering to the principle of putting prevention and comprehensive management. In the same vein, a target responsibility system for environmental protection must be established, the system of environmental regulations and standards and social supervision mechanisms should be improved, and environmental management in technological innovation should be strengthened while guiding the transformation and upgrading of regional enterprises. Additionally, an advanced technology support system and an evaluation index system should be constructed, supported by vigorous development of the circular economy, construction of environmental infrastructure, reinforcement of the comprehensive prevention and control of urban pollution, and comprehensive enhancement of the quality of the urban atmospheric environment in order to build appropriate ecological service system and enhance the self-purification ability of urban ecosystem by realizing the construction of the urban green space system.

Finally, there is a need to establish a professional team of technological innovation and development and ecological and environmental protection talents. Since talent represents a crucial driving force to promote development, therefore Gansu province should create a special talent team around the ecological environment and technological innovation, performance management, R&D, and maintenance along the ecological environment protection, innovation chain, and its economic impact, in order to ensure that the technological innovation and ecological environment of the coupling and coordinated development level continuously improve and remain in a sound state.

## Supporting information

S1 Data (DOC)
