# Supplementary material for: The characteristics and countermeasures of coupled and coordinated development between technological innovation and ecological environment in China’s Gansu province
Source: PLoS One. 2023 Oct 3;18(10):e0290704. doi: 10.1371/journal.pone.0290704 (PMC10547164; doi:10.1371/journal.pone.0290704)
Supplement: S1 Data — (DOC) [file pone.0290704.s001.doc]

| **First-class indicators;;** | **Secondary indicators** | **Three-level indicators** | **Weight** | **attribute** | **LANZHOU** | | | | | **JIAYUGUAN** | | | | |
| --- | --- | --- | --- | --- | --- | --- | --- | --- | --- | --- | --- | --- | --- | --- |
|  |  |  |  |  | **2015** | **2016** | **2017** | **2018** | **2019** | **2015** | **2016** | **2017** | **2018** | **2019** |
| A1 | B1 | C1 |  | + | 204 | 214 | 220 | 211 | 251 | 10 | 11 | 17 | 9 | 16 |
|  |  | C2 |  | + | 14173 | 14025 | 13435 | 12862 | 27832 | 1292 | 1184 | 546 | 372 | 1996 |
|  |  | C3 |  | + | 405523 | 409508 | 326160 | 523714 | 639219 | 81058 | 106740 | 104221 | 95358 | 99210 |
|  | B2 | C4 |  | + | 40.23 | 48.83 | 56.14 | 62.86 | 67.18 | 4.31 | 4.73 | 5.20 | 0.26 | 0.41 |
|  |  | C5 |  | + | 6.28 | 7.54 | 9.18 | 10.67 | 12.92 | 6.7 | 7.76 | 14.03 | 10.77 | 14.77 |
|  |  | C6 |  | + | 19.15 | 17.10 | 15.18 | 14.2 | 14.6 | 20.1 | 17.75 | 11.53 | 14.0 | 31.7 |
|  | B3 | C7 |  | + | 1.93 | 1.81 | 1.88 | 1.92 | 2.25 | 4.27 | 6.96 | 4.97 | 3.18 | 3.50 |
|  |  | C8 |  | + | 0.98 | 0.98 | 1.0 | 1.03 | 1.08 | 0.14 | 0.14 | 0.15 | 0.13 | 0.14 |
|  |  | C9 |  | + | 82.00 | 84 | 83 | 79 | 69 | 0 | 0 | 0 | 1 | 1 |
| A2 | B4 | C10 |  | - | 1.71 | 1.13 | 0.86 | 0.97 | 1.36 | 19.74 | 23.32 | 12.04 | 12.54 | 3.86 |
|  |  | C11 |  | - | 1.97 | 1.48 | 1.45 | 1.36 | 31.75 | 14.43 | 21.56 | 7.41 | 3.86 | 184.43 |
|  |  | C12 |  | - | 607.75 | 291.05 | 320.58 | 416.65 |  | 886.31 | 838.99 | 927.61 | 802.09 |  |
|  | B5 | C13 |  | + | 9.17 | 12.71 | 12.76 | 13.89 | 13.45 | 37.33 | 36.96 | 37.49 | 36.44 | 37.91 |
|  |  | C14 |  | + | 25.8 | 27.4 | 30.93 | 32.03 | 34.35 | 39.5 | 39.2 | 39.41 | 40.01 | 40.27 |
|  |  | C15 |  | + | 98 | 103 | 105 | 105 | 105 | 300 | 314 | 448 | 445 | 444 |
|  | B6 | C16 |  | + | 98.36 | 96.08 | 97.8 | 104 | 112.69 | 7.3 | 6.7 | 6.7 | 8 | 9.94 |
|  |  | C17 |  | + | 94 | 81 | 76 | 83 | 88 | 1 | 1 | 1 | 2 | 3 |
|  |  | C18 |  | + | 598.41 | 280.72 | 282.24 | 402.67 | 512.12 | 428.47 | 482.86 | 583.08 | 515.51 | 524.13 |

| **First-class indicators;;** | **Secondary indicators** | **Three-level indicators** | **Weight** | **attribute** | **JINCHANG** | | | | | **BAIYIN** | | | | |
| --- | --- | --- | --- | --- | --- | --- | --- | --- | --- | --- | --- | --- | --- | --- |
|  |  |  |  |  | **2015** | **2016** | **2017** | **2018** | **2019** | **2015** | **2016** | **2017** | **2018** | **2019** |
| A1 | B1 | C1 |  | + | 12 | 15 | 17 | 16 | 23 | 27 | 36 | 27 | 30 | 39 |
|  |  | C2 |  | + | 1089 | 1055 | 1699 | 695 | 1562 | 769 | 907 | 936 | 812 | 2286 |
|  |  | C3 |  | + | 110497 | 102770 | 94989 | 45095 | 45220 | 39871 | 42340 | 42340 | 52715 | 65915 |
|  | B2 | C4 |  | + | 3.00 | 3.41 | 3.64 | 4.28 | 5.52 | 2.68 | 3.44 | 3.95 | 5.50 | 6.23 |
|  |  | C5 |  | + | 6.8 | 7.5 | 8.64 | 9.44 | 11.67 | 8.09 | 9.44 | 11.02 | 9.78 | 10.82 |
|  |  | C6 |  | + | 33.12 | 37.95 | 41.54 | 31.7 | 33.4 | 13.15 | 13.98 | 13.04 | 15.6 | 15.1 |
|  | B3 | C7 |  | + | 4.92 | 4.95 | 4.33 | 1.71 | 1.33 | 0.92 | 0.96 | 0.97 | 1.03 | 1.36 |
|  |  | C8 |  | + | 0.07 | 0.07 | 0.07 | 0.07 | 0.09 | 0.02 | 0.02 | 0 | 0.02 | 0.03 |
|  |  | C9 |  | + | 0 | 0 | 2 | 2 | 2 | 2 | 2 | 3 | 3 | 2 |
| A2 | B4 | C10 |  | - | 4.17 | 3.30 | 2.38 | 1.91 | 4.55 | 2.31 | 2.17 | 1.60 | 2.86 | 0.91 |
|  |  | C11 |  | - | 9.03 | 7.47 | 7.00 | 4.55 | 24.29 | 1.31 | 0.90 | 0.83 | 0.91 | 8.54 |
|  |  | C12 |  | - | 1229.23 | 1091.14 | 1150.88 | 1198.85 |  | 526.46 | 624.65 | 505.88 | 697.97 |  |
|  | B5 | C13 |  | + | 21.01 | 22.86 | 24.98 | 27.71 | 24.41 | 9.71 | 9.51 | 9.51 | 9.49 | 11.54 |
|  |  | C14 |  | + | 36.3 | 36.7 | 37.08 | 37.62 | 39.57 | 35.1 | 34.7 | 32.51 | 36.39 | 36.48 |
|  |  | C15 |  | + | 132 | 133 | 133 | 133 | 137 | 77 | 75 | 78 | 75 | 75 |
|  | B6 | C16 |  | + | 9.2 | 9.2 | 5.8 | 5.5 | 6.1 | 15.96 | 15.97 | 15.4 | 13.7 | 11.32 |
|  |  | C17 |  | + | 66 | 18 | 21 | 27 | 33 | 71 | 51 | 68 | 73 | 78 |
|  |  | C18 |  | + | 160.88 | 146.24 | 168.46 | 168.74 | 188.45 | 304.53 | 375.78 | 311.76 | 515.39 | 615.23 |

| **First-class indicators;;** | **Secondary indicators** | **Three-level indicators** | **Weight** | **attribute** | **TIANSHUI** | | | | | **WUWEI** | | | | |
| --- | --- | --- | --- | --- | --- | --- | --- | --- | --- | --- | --- | --- | --- | --- |
|  |  |  |  |  | **2015** | **2016** | **2017** | **2018** | **2019** | **2015** | **2016** | **2017** | **2018** | **2019** |
| A1 | B1 | C1 |  | + | 32 | 43 | 46 | 39 | 37 | 120 | 118 | 103 | 90 | 115 |
|  |  | C2 |  | + | 1535 | 1884 | 1729 | 1318 | 2539 | 1580 | 1363 | 1243 | 1221 | 2211 |
|  |  | C3 |  | + | 31692 | 34635 | 35059 | 81410 | 76667 | 24677 | 25808 | 18714 | 21994 | 34895 |
|  | B2 | C4 |  | + | 18.57 | 20.37 | 22.46 | 25.01 | 27.56 | 8.45 | 9.11 | 10.21 | 11.27 | 13.12 |
|  |  | C5 |  | + | 6.06 | 8.57 | 9.19 | 9.86 | 11.84 | 6.66 | 10.04 | 13.73 | 14.82 | 13.61 |
|  |  | C6 |  | + | 4.12 | 4.64 | 4.25 | 7.6 | 8.9 | 2.23 | 2.77 | 2.27 | 2.1 | 2.5 |
|  | B3 | C7 |  | + | 0.57 | 0.59 | 0.72 | 1.25 | 1.21 | 0.59 | 0.56 | 0.52 | 0.47 | 0.71 |
|  |  | C8 |  | + | 0.1 | 0.1 | 0.1 | 0.1 | 0.11 | 0.09 | 0.09 | 0.08 | 0.08 | 0.1 |
|  |  | C9 |  | + | 9 | 11 | 10 | 10 | 10 | 5 | 11 | 11 | 13 | 30 |
| A2 | B4 | C10 |  | - | 0.48 | 0.39 | 0.51 | 0.50 | 0.52 | 0.77 | 0.31 | 0.40 | 0.52 | 0.50 |
|  |  | C11 |  | - | 0.89 | 0.56 | 0.48 | 0.52 | 1.18 | 2.27 | 0.74 | 0.49 | 0.50 | 1.74 |
|  |  | C12 |  | - | 69.22 | 47.51 | 28.16 | 24.86 | / | 65.75 | 48.70 | 57.30 | 75.86 | / |
|  | B5 | C13 |  | + | 9.84 | 9.89 | 9.92 | 9.95 | 10.12 | 14.67 | 14.96 | 23.06 | 8.46 | 9.98 |
|  |  | C14 |  | + | 38.1 | 38.4 | 38.68 | 38.95 | 39.49 | 23.6 | 25.8 | 26.08 | 31.03 | 36.03 |
|  |  | C15 |  | + | 41 | 41 | 38 | 40 | 39.31 | 47 | 47 | 46 | 44 | 43 |
|  | B6 | C16 |  | + | 21.78 | 22.1 | 22.1 | 24.7 | 23.43 | 18 | 18 | 21.9 | 23.7 | 14.86 |
|  |  | C17 |  | + | 47 | 37 | 35 | 38 | 41 | 29 | 17 | 23 | 27 | 32 |
|  |  | C18 |  | + | 58.46 | 34.07 | 20.46 | 14.57 | 20.12 | 59.37 | 43.24 | 49.03 | 56.37 | 66.32 |

| **First-class indicators;;** | **Secondary indicators** | **Three-level indicators** | **Weight** | **attribute** | **ZHANGYE** | | | | | **PINGLIANG** | | | | |
| --- | --- | --- | --- | --- | --- | --- | --- | --- | --- | --- | --- | --- | --- | --- |
|  |  |  |  |  | **2015** | **2016** | **2017** | **2018** | **2019** | **2015** | **2016** | **2017** | **2018** | **2019** |
| A1 | B1 | C1 |  | + | 113 | 97 | 109 | 99 | 113 | 11 | 11 | 10 | 11 | 18 |
|  |  | C2 |  | + | 1221 | 1281 | 1048 | 1189 | 2341 | 404 | 336 | 368 | 372 | 567 |
|  |  | C3 |  | + | 44122 | 51023 | 47090 | 51461 | 58219 | 5505 | 4509 | 1475 | 7937 | 8584 |
|  | B2 | C4 |  | + | 13.20 | 16.06 | 20.21 | 22.67 | 25.56 | 12.35 | 13.63 | 4.96 | 3.15 | 3.79 |
|  |  | C5 |  | + | 5.58 | 8.9 | 15.18 | 23.34 | 15.13 | 5.78 | 7.57 | 10.9 | 10.61 | 12.2 |
|  |  | C6 |  | + | 5.15 | 5.73 | 5.98 | 5.0 | 5.4 | 1.21 | 1.30 | 0.98 | 2.3 | 1.4 |
|  | B3 | C7 |  | + | 1.18 | 1.28 | 1.31 | 1.26 | 1.30 | 0.16 | 0.12 | 0.10 | 0.20 | 0.19 |
|  |  | C8 |  | + | 0.15 | 0.15 | 0.15 | 0.14 | 0.14 | 0.02 | 0.02 | 0.03 | 0.05 | 0.06 |
|  |  | C9 |  | + | 5 | 6 | 6 | 6 | 6 | 6 | 9 | 9 | 10 | 12 |
| A2 | B4 | C10 |  | - | 2.03 | 0.76 | 0.64 | 0.67 | 0.98 | 3.47 | 2.30 | 2.44 | 2.05 | 1.85 |
|  |  | C11 |  | - | 5.13 | 2.26 | 1.1 | 0.98 | 1.86 | 5.43 | 1.85 | 2.20 | 1.85 | 1.89 |
|  |  | C12 |  | - | 911.93 | 964.03 | 746.24 | 336.26 | / | 491.81 | 409.08 | 375.52 | 375.68 | / |
|  | B5 | C13 |  | + | 38.11 | 45.17 | 51.66 | 20.66 | 22.47 | 7.92 | 8.35 | 11.25 | 12.89 | 12.93 |
|  |  | C14 |  | + | 30.7 | 38.8 | 53.71 | 35.69 | 35.82 | 35.7 | 37.1 | 39.36 | 39.44 | 39.54 |
|  |  | C15 |  | + | 73 | 74 | 71 | 76 | 75 | 47 | 48 | 48 | 47 | 47 |
|  | B6 | C16 |  | + | 9.89 | 10.7 | 9.2 | 11.2 | 11.88 | 13.8 | 13.85 | 10.1 | 10.1 | 11.8 |
|  |  | C17 |  | + | 74 | 57 | 62 | 72 | 78 | 62 | 68 | 68 | 72 | 76 |
|  |  | C18 |  | + | 691.19 | 758.18 | 580.84 | 98.47 | 125.45 | 309.29 | 337.94 | 223.32 | 205.55 | 215.14 |

| **First-class indicators;;** | **Secondary indicators** | **Three-level indicators** | **Weight** | **attribute** | **JIUQUAN** | | | | | **QINGYANG** | | | | |
| --- | --- | --- | --- | --- | --- | --- | --- | --- | --- | --- | --- | --- | --- | --- |
|  |  |  |  |  | **2015** | **2016** | **2017** | **2018** | **2019** | **2015** | **2016** | **2017** | **2018** | **2019** |
| A1 | B1 | C1 |  | + | 73 | 83 | 46 | 31 | 51 | 47 | 24 | 16 | 11 | 13 |
|  |  | C2 |  | + | 1621 | 1852 | 1127 | 2007 | 1806 | 1572 | 1202 | 869 | 706 | 1148 |
|  |  | C3 |  | + | 51222 | 60882 | 20854 | 62142 | 41342 | 23464 | 19364 | 8252 | 4837 | 7783 |
|  | B2 | C4 |  | + | 19.44 | 22.33 | 24.32 | 27.10 | 24.41 | 2.15 | 2.31 | 3.01 | 4.58 | 8.83 |
|  |  | C5 |  | + | 7.2 | 8.81 | 13.73 | 11.08 | 13.41 | 6.73 | 8.33 | 12.93 | 9.62 | 14.9 |
|  |  | C6 |  | + | 5.1 | 5.54 | 2.27 | 5.6 | 3.6 | 2.31 | 2.14 | 0.81 | 0.5 | 0.8 |
|  | B3 | C7 |  | + | 0.94 | 1.05 | 0.49 | 1.04 | 0.67 | 0.39 | 0.32 | 0.16 | 0.07 | 0.10 |
|  |  | C8 |  | + | 0.07 | 0.08 | 0.08 | 0.09 | 0.11 | 0.06 | 0.06 | 0.07 | 0.07 | 0.07 |
|  |  | C9 |  | + | 6 | 13 | 13 | 11 | 9 | 4 | 5 | 5 | 5 | 5 |
| A2 | B4 | C10 |  | - | 0.63 | 0.45 | 0.62 | 0.67 | 0.58 | 0.19 | 0.22 | 0.31 | 0.27 | 0.24 |
|  |  | C11 |  | - | 1.74 | 0.88 | 0.67 | 0.58 | 8.32 | 0.41 | 0.41 | 0.47 | 0.24 | 1.33 |
|  |  | C12 |  | - | 312.27 | 144.64 | 303.65 | 320.83 | / | 16.06 | 13.18 | 21.07 | 17.53 | / |
|  | B5 | C13 |  | + | 11.53 | 11.45 | 11.63 | 11.75 | 11.72 | 7.33 | 7.48 | 7.24 | 11.80 | 11.79 |
|  |  | C14 |  | + | 37.4 | 37.1 | 37.21 | 37.22 | 37.60 | 33.4 | 33.8 | 30.42 | 31.90 | 32.04 |
|  |  | C15 |  | + | 67 | 65 | 65 | 64 | 64 | 32 | 31 | 30 | 29 | 28 |
|  | B6 | C16 |  | + | 12 | 12.33 | 12.1 | 13 | 15.1 | 15.7 | 12.45 | 12.5 |  | 15.35 |
|  |  | C17 |  | + | 42 | 44 | 53 | 52 | 56 | 29 | 17 | 14 | 20 | 25 |
|  |  | C18 |  | + | 355.71 | 75.51 | 120.34 | 91.55 | 156.21 | 15.67 | 13.18 | 18.70 | 15.83 | 17.45 |

| **First-class indicators;;** | **Secondary indicators** | **Three-level indicators** | **Weight** | **attribute** | **DINGXI** | | | | | **LONGNAN** | | | | |
| --- | --- | --- | --- | --- | --- | --- | --- | --- | --- | --- | --- | --- | --- | --- |
|  |  |  |  |  | **2015** | **2016** | **2017** | **2018** | **2019** | **2015** | **2016** | **2017** | **2018** | **2019** |
| A1 | B1 | C1 |  | + | 12 | 17 | 23 | 17 | 20 | 19 | 17 | 16 | 12 | 13 |
|  |  | C2 |  | + | 179 | 360 | 280 | 304 | 532 | 99 | 170 | 200 | 168 | 654 |
|  |  | C3 |  | + | 2155 | 5741 | 7513 | 12278 | 9026 | 2859 | 4574 | 4631 | 5364 | 6463 |
|  | B2 | C4 |  | + | 2.20 | 2.58 | 3.88 | 5.47 | 6.23 | 2.66 | 2.67 | 3.29 | 6.75 | 5.22 |
|  |  | C5 |  | + | 6.59 | 7.51 | 9.11 | 9.18 | 10.14 | 5.65 | 10.03 | 14.29 | 14.26 | 13.15 |
|  |  | C6 |  | + | 1.11 | 0.63 | 1.13 | 2.8 | 1.3 | 2.52 | 2.84 | 1.14 | 0.7 | 1.0 |
|  | B3 | C7 |  | + | 0.07 | 0.17 | 0.26 | 0.34 | 0.22 | 0.09 | 0.13 | 0.15 | 0.14 | 0.15 |
|  |  | C8 |  | + | 0.02 | 0.01 | 0.02 | 0.02 | 0.02 | 0.02 | 0.02 | 0.02 | 0.02 | 0.03 |
|  |  | C9 |  | + | 4 | 4 | 4 | 4 | 4 | 4 | 4 | 4 | 4 | 4 |
| A2 | B4 | C10 |  | - | 1.28 | 1.0 | 1.61 | 1.32 | 0.35 | 1.02 | 0.87 | 0.49 | 0.34 | 0.75 |
|  |  | C11 |  | - | 0.81 | 0.61 | 0.44 | 0.35 | 0.58 | 3.85 | 0.75 | 1.17 | 0.75 | 0.5 |
|  |  | C12 |  | - | 21.54 | 23.57 | 31.48 | 33.88 | / | 557.33 | 500.40 | 488.79 | 540.89 | / |
|  | B5 | C13 |  | + | 16.39 | 16.56 | 16.56 | 16.85 | 16.85 | 5.03 | 5.71 | 5.99 | 6.15 | 13.42 |
|  |  | C14 |  | + | 25.2 | 25.2 | 25.66 | 26.40 | 26.97 | 8.4 | 10.1 | 14.18 | 14.37 | 33.04 |
|  |  | C15 |  | + | 28 | 27 | 25 | 25 | 24 | 13 | 12 | 15 | 14 | 14 |
|  | B6 | C16 |  | + | 6.5 | 6.5 | 6.5 | 13.1 | 5.41 | 4.79 | 4.96 | 5.5 | 6 | 6.58 |
|  |  | C17 |  | + | 40 | 53 | 39 | 49 | 58 | 80 | 46 | 53 | 44 | 46 |
|  |  | C18 |  | + | 19.47 | 21.19 | 23.26 | 33.80 | 41.12 | 18.58 | 9.35 | 9.53 | 23.26 | 45.56 |

| **First-class indicators;;** | **Secondary indicators** | **Three-level indicators** | **Weight** | **attribute** | **LINXIA** | | | | | **GANNAN** | | | | |
| --- | --- | --- | --- | --- | --- | --- | --- | --- | --- | --- | --- | --- | --- | --- |
|  |  |  |  |  | **2015** | **2016** | **2017** | **2018** | **2019** | **2015** | **2016** | **2017** | **2018** | **2019** |
| A1 | B1 | C1 |  | + | 6 | 4 | 10 | 10 | 18 | 7 | 5 | 6 | 6 | 5 |
|  |  | C2 |  | + | 146 | 93 | 181 | 104 | 399 | 84 | 50 | 76 | 83 | 174 |
|  |  | C3 |  | + | 3769 | 1307 | 2139 | 4968 | 8782 | 789 | 651 | 236 | 1264 | 1120 |
|  | B2 | C4 |  | + | 0.59 | 0.65 | 0.92 | 1.12 | 1.22 | 0.47 | 0.69 | 0.76 | 0.85 | 1.14 |
|  |  | C5 |  | + | 5.61 | 5.18 | 10.11 | 12.21 | 19.95 | 8.61 | 10.62 | 6.20 | 9.57 | 10.11 |
|  |  | C6 |  | + | 0.51 | 0.44 | 0.34 | 0.6 | 0.9 | 0.01 | 0.02 | 0.02 | 0.1 | 0.1 |
|  | B3 | C7 |  | + | 0.18 | 0.06 | 0.12 | 0.19 | 0.29 | 0.06 | 0.05 | 0.05 | 0.08 | 0.05 |
|  |  | C8 |  | + | 0 | 0 | 0.02 | 0.02 | 0.02 | 0.15 | 0.15 | 0.14 | 0.14 | 0.14 |
|  |  | C9 |  | + | 1 | 1 | 1 | 1 | 1 | 4 | 4 | 4 | 4 | 4 |
| A2 | B4 | C10 |  | - | 0.82 | 0.62 | 0.42 | 0.33 | 1.03 | 0.33 | 0.23 | 0.18 | 0.11 | 0.38 |
|  |  | C11 |  | - | 3.06 | 0.93 | 1.35 | 1.03 | 1.38 | 0.24 | 0.12 | 0.08 | 0.38 | 4.17 |
|  |  | C12 |  | - | 14.38 | 9.81 | 8.95 | 9.18 | / | 98.66 | 62.70 | 52.50 | 92.00 | / |
|  | B5 | C13 |  | + | 5.10 | 5 | 5.26 | 5.16 | 5.33 | 6.98 | 6.98 | 8.61 | 8.54 | 17.02 |
|  |  | C14 |  | + | 14.4 | 14.5 | 11.98 | 11.98 | 31.66 | 6.5 | 9.3 | 9.27 | 9.13 | 38.17 |
|  |  | C15 |  | + | 36 | 36 | 33 | 27 | 27 | 48 | 46 | 41 | 38 | 43 |
|  | B6 | C16 |  | + | 18.52 | 17.18 | 17.4 | 16.5 | 17.2 | 3.32 | 3.33 | 3.1 | 3.3 | 5.55 |
|  |  | C17 |  | + | 26 | 28 | 30 | 38 | 44 | 2 | 9 | 12 | 10 | 13 |
|  |  | C18 |  | + | 14.38 | 9.81 | 8.95 | 9.18 | 10.12 | 36.94 | 32.16 | 27.38 | 28.36 | 30.54 |
